# Risk communication during COVID-19: A descriptive study on familiarity with, adherence to and trust in the WHO preventive measures

**DOI:** 10.1371/journal.pone.0250872

**Published:** 2021-04-29

**Authors:** Nirosha Elsem Varghese, Iryna Sabat, Sebastian Neumann-Böhme, Jonas Schreyögg, Tom Stargardt, Aleksandra Torbica, Job van Exel, Pedro Pita Barros, Werner Brouwer

**Affiliations:** 1 Centre for Research on Health and Social Care Management, CERGAS, Bocconi University, Milan, Italy; 2 Nova School of Business and Economics, Lisbon, Portugal; 3 Erasmus School of Health Policy & Management, Erasmus University Rotterdam, Rotterdam, The Netherlands; 4 Hamburg Center for Health Economics, University of Hamburg, Hamburg, Germany; 5 Erasmus School of Economics, Erasmus University Rotterdam, Rotterdam, The Netherlands; University of Haifa, ISRAEL

## Abstract

**Background:**

Risk communication is a key component of public health interventions during an outbreak. As the coronavirus pandemic unfolded in late 2019, the World Health Organization (WHO) was at the forefront in the development of risk communication strategies. The WHO introduced a range of activities with the purpose of enabling the public to avail verified and timely information on COVID-19 prevention behaviors. Given the various WHO activities to protect the public health during COVID-19, it is important to investigate the extent of familiarity and uptake of the WHO recommendations among the public during the first wave of the pandemic.

**Methods:**

To do this, we conducted a large-scale Pan-European survey covering around 7500 individuals that are representative of populations from seven European countries, collected online during April 2-April 15, 2020. We use descriptive statistics including proportions and correlations and graphical representations such as bar charts to analyze and display the data.

**Results:**

Our findings suggest that information from the WHO in the context of COVID-19 is well trusted and acted upon by the public. Overall familiarity and adherence were quite high in most countries. Adherence was higher for social distancing recommendations compared to hygiene measures. Familiarity and adherence were higher among older, female, and highly educated respondents. However, country level heterogeneities were observed in the level of trust in information from the WHO, with countries severely affected by the pandemic reporting lower levels of trust.

**Conclusion:**

Our findings call for efforts from health authorities to get regular feedback from the public on their familiarity and compliance with recommendations for preventive measures at all stages of the pandemic, to further develop and adapt risk communication as the pandemic evolves.

## Introduction

Risk communication is key to improving familiarity with and adherence to preventive measures, in normal times but also particularly during health emergencies. Failure to communicate the right message effectively can result in loss of trust, damage to the economy and loss of lives [1]. For risk communication to be effective, risk messages have to be shared with the public in an openly and timely manner, so as to reduce the knowledge gap and to convince the public to adjust their behavior during a crisis [2]. In addition to disseminating recommendations that are easy for the public to understand and comply with, trust in the source of the message is important for an effective risk communication [1, 3].

The World Health Organization (WHO) has been in the frontline in its operations to contain and mitigate the spread of the COVID-19 pandemic. The WHO is a key player in disseminating up to date information and recommendations on COVID-19 preventive behaviors to the public [4]. With a physical presence in 149 countries, these recommendations are also adapted to national and local considerations, thereby setting the WHO protocol as a foundation for further containment strategies at various levels of government [5, 6].

As the coronavirus pandemic unfolded in late 2019, the WHO was quick to realize the need for a tailored risk communication strategy. The WHO Information Network for Epidemics (or EPI-WIN) was introduced when COVID-19 was declared a public emergency of international concern on 30^th^ January 2020 [7]. EPI-WIN provides customized information and guidance to specific target groups in addition to fighting the ‘infodemic’ [7]. For example, this involved increasing the public awareness on preventive measures against COVID-19 through easy to understand behavioral messages using infographics and videos on the WHO website. EPI-WIN also guides national governments in risk communication and community engagement according to the transmission scenario of each country with the purpose of developing, implementing, and monitoring a communication plan that can help protect the public health during the health crisis [8]. Another such WHO and national government collaboration in risk communication is the Global Outbreak Alert and Response Network (GOARN), a network of 250 technical institutes across the globe that has been actively involved in co-creating and co-implementing risk communication messages so as to adapt to the local context [9].

Additionally, the WHO undertook a range of other innovative steps to improve risk communication during this pandemic. They teamed up with social media companies and Google to ensure that any search queries related to COVID-19 directs the user to the WHO pages [10]. The WHO introduced an online training course on COVID-19 and collaborated with celebrities on the safe hands challenge to demonstrate hand hygiene on social media [11, 12]. Given all the actions undertaken by the WHO to promote public awareness on COVID-19, it seems important to investigate the familiarity of the public with the WHO recommended preventive measures, whether familiarity translates into adherence to these measures, and the role of trust in the information in this relationship.

## Materials and methods

We use individual level data covering 7000 respondents representative of the adult population (aged 18 and above) in seven European countries: Denmark, France, Germany, Italy, Portugal, the Netherlands, and the UK. The online survey was conducted during April 2–15, 2020 by the market research company Dynata (https://www.dynata.com). To maximize reach and capacity, the respondents were recruited using variety of contact methods (such as websites, emails, social media influencers, TV ads, loyalty partnerships and so on) which builds into a combined panel which is more representative of the offline population. Project details are not included in the invite to reduce self-selection bias. The questionnaire was initially developed in English by the authors of the study and was then translated and adapted to country specific context by native speakers. The questionnaire was first administered as a pilot to collect 10% of the sample which was included to the final sample. Potential participants go through an initial eligibility check using personal information (if available) and other screening questions. Upon informed consent, data was collected from 1000 respondents’ in each country representative of the national population in terms of region, age, gender, and education. Representativeness of the sample was achieved by using quotas for the demographic characteristics based on the national census statistics. Quality checks were carried out on the final sample to eliminate and replace any speeders (below one-third of median time duration taken to complete the survey), slow respondents (above 95^th^ percentile of time duration) and bad responses (answers with no logical consistency or straight liners). Upon completion of the survey, panelists received incentives which they could redeem for a range of gifts, charitable contribution or other services.

As part of a larger survey, respondents were asked about their familiarity with the preventive measures recommended by the WHO, their adherence to these measures, and their trust in the information from the WHO. Socio-demographic variables used in our study include age, gender and level of education (low, medium and high). Level of education was defined based on country specific education system and was provided to us directly by Dynata (See S1 Table). We also collect information on household composition and consider a household to be vulnerable if respondents report having children, disabled, family members with diagnosed chronic medical conditions or elderly at home. The core questions in the survey used for this study can be found in S1 Appendix. Summary statistics including percentages and Spearman’s rank correlations were used to analyze the data. The statistical significance for difference in proportions between groups was tested using Chi-squared test. Additionally, we use graphical representations and simple ranking for summarizing results. To investigate the independent relationship between two variables after partialling out potential confounders, we use multivariate non-linear regressions. Statistical analyses were performed on STATA 15 (STATA Corp, College Station, TX, US).

We also use external country-specific data on the severity of the pandemic and stringency of containment measures to present these country specific differences as a potential factor influencing the relationship between familiarity, trust and adherence. Data on COVID-19 prevalence and COVID-19 attributed deaths per 1 million people are reported by the European Centre for Disease Prevention and Control [13]. To assess the degree of strictness of containment policies in each country, we use the COVID-19 Government Response Stringency Index introduced by the Oxford University and measured on a range from 0–100, with higher values indicating stricter measures [14]. Familiarity and adherence to preventive measures may depend on the pandemic situation of the country and the governmental response at the time of survey. Country-specific data on COVID-19 death (and prevalence) around the time of the survey show a north-south divide in the development of the pandemic with a higher number of COVID-19 deaths being recorded in Italy and France followed by the UK [13]. The timing and stringency of the containment measures also varied across the countries. Italy, France and Portugal imposed strict containment policies compared to other countries included in the survey [14]. Strict measures were implemented in all countries displaying a higher severity of COVID-19 pandemic except for the UK where the government response was less strict in response to the epidemiological crisis faced by the country [13, 14]. To highlight these variations as a means to further understand the country level differences in public response to WHO recommendations, we provide a timely description of the epidemiological situation and stringency of containment policies in each country (Table 1).

**Table 1:** Relative ranking of countries according to the severity of the pandemic and stringency of containment measures.

	Rank		
	Cases	Deaths	Stringency index
Italy	1	1	1
France	4	2	2
UK	6	3	5
Netherlands	5	4	4
Portugal	2	5	3
Denmark	7	6	6
Germany	3	7	7

Note 1: Cases and deaths refer to total confirmed COVID-19 cases and COVID-19 attributed deaths (per 1 million people). Stringency index refers to degree of strictness of government response to COVID-19 (measured between 0–100).

Note 2: The rankings are based on data collected from the source [13, 14] as of April 12, 2020

### Ethics approval and consent to participate

Ethical approval for this study was provided by the University of Hamburg, Germany under the umbrella project “Countering COVID-19: A European survey on the acceptability of and commitment to preventive measures”. Subject recruitment and payment were done through the Agency Dynata. Proprietary panels used double opt-in recruitment and a written informed consent was obtained from individual participants by Dynata. Confidentiality and anonymity of the participants were ensured by issuing a unique identifier to each respondent. Study participants were informed about their freedom to opt-out of the study at any point of time. No patients are involved in this research and we strictly followed the RESPECT Code of practice for the conduct of socio-economic research in Europe. Since no patients were involved, we were not required to obtain ethical approval from all countries. Prior to the start of the project, the first author also completed the web-based course "Protecting Human Research Participants Online Training" on topics including human subject protections, ethical issues associated with human subject research, and current regulatory and guidance information.

## Results

### Familiarity with the WHO recommendations

In the wake of the COVID-19 crisis, the WHO put forward six basic preventive measures to help contain and mitigate the spread of coronavirus. The recommendations were first released on January 10, 2020 on the WHO website, around 11 weeks prior to the release of our survey [7]. The recommendations included timely and easy-to-understand measures such as regularly washing hands with soap for at least 20 seconds, covering nose and mouth while coughing or sneezing, keeping a social distance of at least 1 meter, avoid shaking hands, hugging or kissing when greeting others, using alcohol-based hand rub and avoid touching nose, eyes and mouth. In our survey, respondents were shown the graphic presentation of the six measures that was used for communication in their country and asked to rate their familiarity with the measures on a scale from “not at all familiar” (1) to “very familiar” (5). Respondents reporting a score of 4 (moderately familiar) or 5 (very familiar) are classified as being familiar with the WHO recommendations.

**Fig 1:**
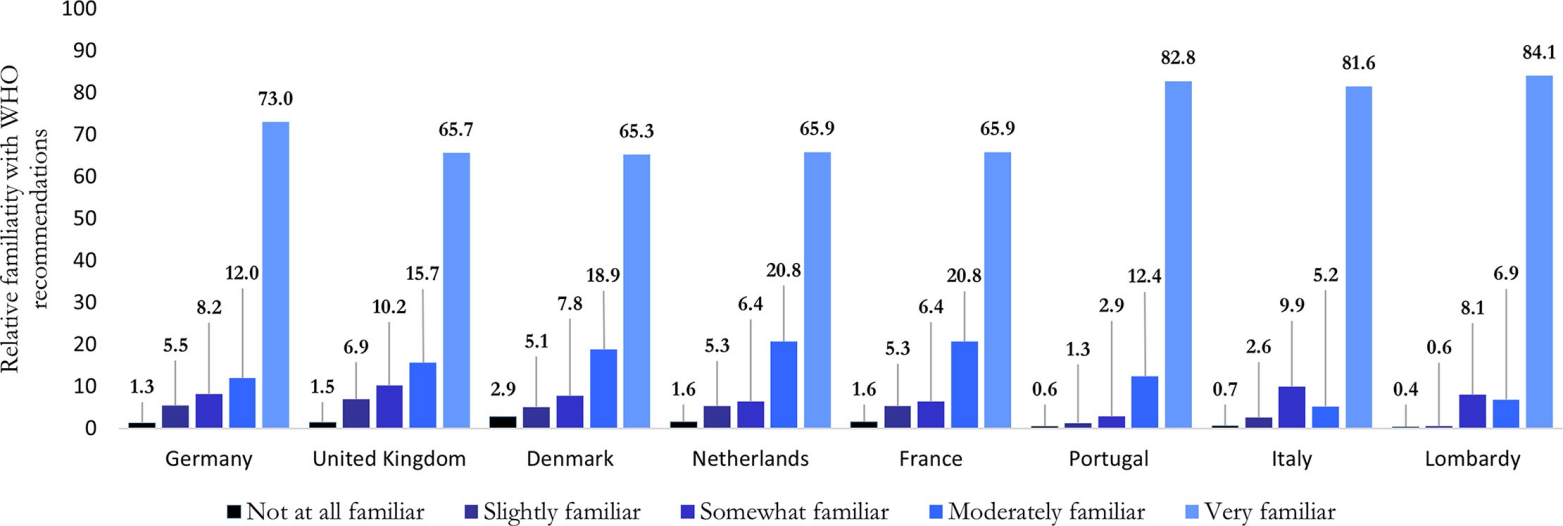
Familiarity with the WHO recommendations, by country.

On average 86.3% of the respondents reported being familiar with the WHO recommended measures. Looking at country level variations (Fig 1), we see that the proportion of respondents who reported being familiar with the recommendations was the highest in Portugal (95.2%) and the lowest in the United Kingdom (81.4%; p<0.001). The other countries in the sample reveal similar levels of familiarity (84–87%) with the WHO recommendations. It is noteworthy that in the Lombardy region familiarity was also very high (91.0%), but especially also that the proportion of the population ‘not at all familiar’ (0.4%) or only ‘slightly familiar’ (0.6%) was the lowest.

Across countries, we find a higher proportion of female (88.4% for females vs. 84.0% for males; p<0.001), older (89.5% for 65+ vs. 78.1 for 18–24 yo; p<0.001) and highly educated (87.5% for high/medium vs. 83.6% for low; p<0.001) respondents reporting familiarity with the WHO recommendations. The same patterns are observed within each country as well. Finally, it should be noted that respondents may overstate their familiarity resulting in self-reporting bias.

### Adherence to WHO recommendations on preventive behavior

**Fig 2:**
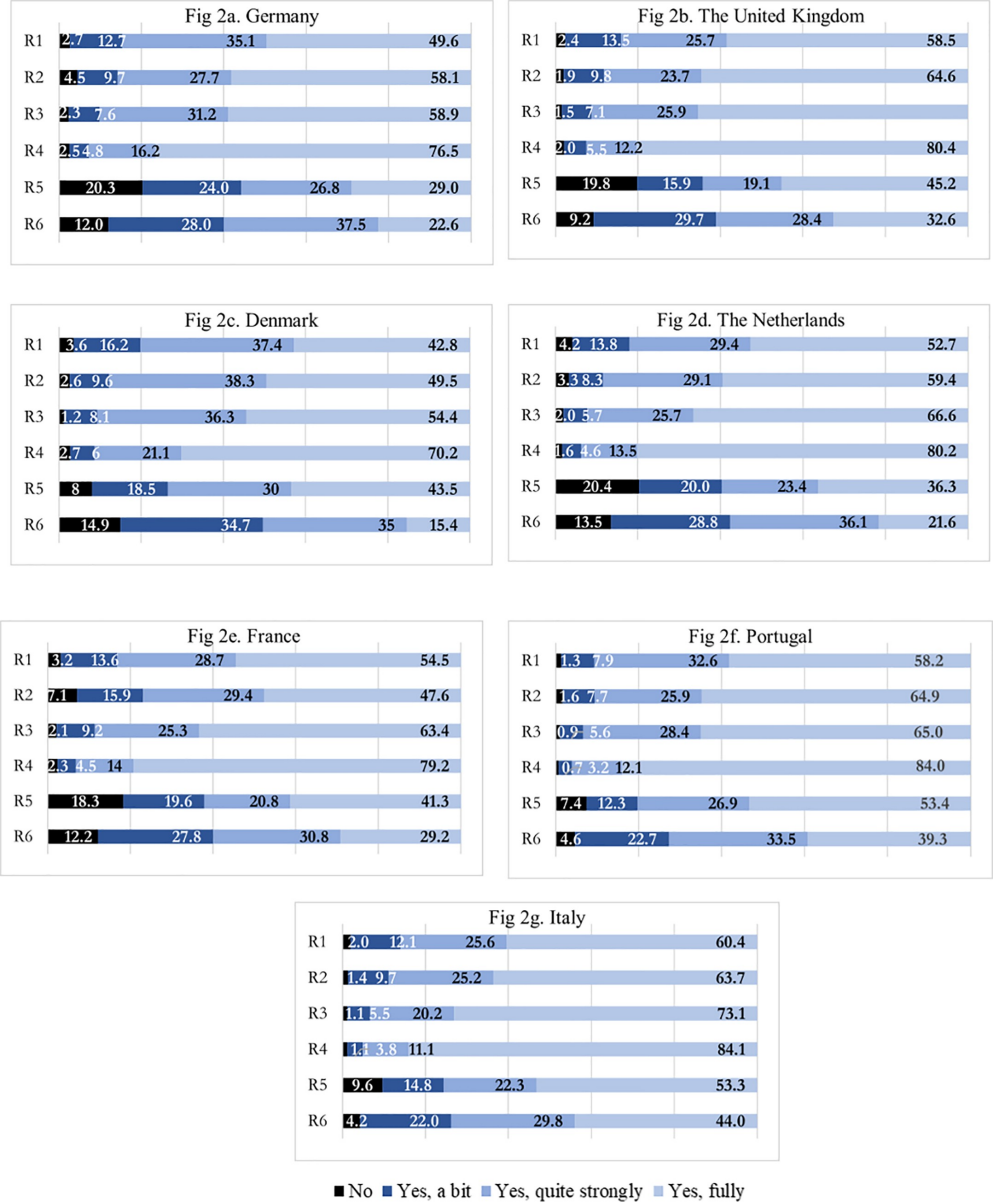
Adherence to WHO recommendations, by country. **R1**: Regularly wash my hands with soap for at least 20 seconds, **R2**: Cover my nose and mouth when coughing or sneezing, **R3**: Keep a distance of at least 1 meter from other people, **R4**: Avoid shaking hands, hugging or kissing when greeting others, **R5**: Use alcohol-based hand rub’ and **R6**: Avoid touching my nose, eyes and mouth.

In our study, we asked respondents to rate their adherence to the six preventive measures over the past four weeks using four levels: no; yes, a bit; yes, quite strongly; yes, fully. We consider respondents to adhere to the recommendations if they reported ‘yes, quite strongly’ or ‘yes, fully’ to each of the six recommendations. Overall, we see that 92.1% of the respondents reported to have adopted the WHO recommendations. Avoiding physical contact by not shaking hands, kissing or hugging when meeting others (93.6%) and keeping a social distance of 1 meter (91.5%) had an overall higher adherence rate, whereas using an alcohol-based hand rub (67.5%) and avoiding touching nose, eyes and mouth (62.4%) had the lowest rates of adherence (Fig 2).

Comparing countries, Portugal and Italy perform best in adhering to all the WHO recommendations whereas France and Denmark perform the worst (Table 2). The difference between the top and worst adhering countries for each WHO recommendation is statistically significant (p<0.001).

**Table 2:** Rank of WHO recommendations in the order of their relative adherence.

Rank	WHO recommendations	Top adherers	Worst adherers
1	Avoid shaking hands, hugging or kissing when greeting others.	Portugal, Italy	Denmark
2	Keep a distance of at least 1 meter from other people.	Portugal, Italy	France
3	Cover my nose and mouth when coughing or sneezing.	Portugal	France
4	Regularly wash my hands with soap for at least 20 seconds.	Portugal	Denmark
5	Use alcohol-based hand rub.	Portugal	Germany
6	Avoid touching my nose, eyes and mouth.	Portugal, Italy	Denmark

Finally, the proportion reporting adherence is higher among female (94.0% for females vs. 90.0% for males; p<0.001) and older (95.1% for 65+ vs. 87.5% for 18–24 yo; p<0.001). If we consider 25–64 years as our reference, we find that respondents aged 18–24 are less likely to comply (87.5% vs. 91.9%; p<0.001) whereas respondents aged 65 and above are more likely to comply (95.1% vs. 91.9%; p<0.001). Respondents reporting to ‘adhere fully’ are higher among those with high/medium level of education (51.0%) compared to low (46.5%; p<0.001) and also among those who have family members that are vulnerable, such as elderly and those with additional comorbidities (49.7%), compared to those who indicate they do not have vulnerable family members (45.6%, p<0.001). Similar results are observed within countries except for levels of education which does not follow a consistent pattern in all countries.

### Perception of adherence to the WHO recommendations by others

We also asked respondents if, according to them, others in the community adhered to the six WHO recommendations these days. The possible responses are: no; yes, a bit; yes, quite strongly; yes, fully. We take a mean of the reported responses across the six recommendations to compute an overall score of respondent’s perception of adherence by others. We then consider respondents to adhere to the recommendations if they reported ‘yes, quite strongly’ or ‘yes, fully’ to each of the six recommendations.

Overall, the proportion of respondents who report that others adhere to the WHO recommendations is 81.3%, which is considerably lower than their own adherence (92.2%). This difference is highest in the UK (19% points difference; p<0.001) and the lowest in the Netherlands (3%; p = 0.022) and in France (1%; p<0.001). Also, it should be noted that respondents could be overstating their own adherence to avoid judgement whereas adherence estimates of others could be a truer estimate of their own actual adherence.

### Trust in information from the WHO

Furthermore, we asked respondents to rate their level of trust in information from the WHO in the context of COVID-19 on a scale “no trust at all” (1) to “trust very much” (5). Respondents reporting a score of 4 or 5 are classified as having trust in the information from the WHO, and those with a score of 1 or 2 as having no trust in this information. We find that on average 59.8% of the respondents from the countries included in this study trust the information on COVID-19 from the WHO, while 15.5% do not trust this information.

Table 3 shows the proportion of respondents in each country reporting trust or no trust in the information from the WHO and the relative ranking among the countries in terms of trust. Marked differences in trust is observed between the countries. In particular, we find that trust is highest in Denmark and Netherlands and the lowest in France with the differences between countries being statistically significant (p<0.001). Similarly, Denmark scores the lowest on distrust whereas France scores the highest followed by Italy, two countries that were the most impacted by the COVID-19 (p<0.001).

**Table 3:** Relative ranking of countries according to the proportion of respondents who trust and distrust information from the WHO.

**Country**	**Trust (%)**	**Rank**
Denmark	64.7	1
Portugal	64.5	2
UK	61.6	3
Italy	60.9	4
Netherlands	59.7	5
Germany	56.8	6
France	49.9	7
**Country**	**Distrust (%)**	**Rank**
France	22.6	1
Italy	16.4	2
Germany	15.7	3
UK	14.1	4
Netherlands	14.0	5
Portugal	13.0	6
Denmark	12.7	7

### Do familiarity and trust breed adherence?

We present evidence suggesting that familiarity and trust could be driving factors for adherence. First, looking at the piecewise relationship between familiarity and adherence, we find that overall familiarity with the six WHO recommendations is significantly correlated with adherence to these recommendations (Table 4), especially for hygiene measures (R1, R2) and avoiding physical contact (R3, R4).

**Table 4:** Correlations between level of familiarity and level of adherence.

Adherence	Familiarity							
	Germany	United Kingdom	Denmark	Netherlands	France	Portugal	Italy	Overall
R1	0.303*	0.254*	0.255*	0.245*	0.239*	0.142*	0.218*	0.247*
R2	0.314*	0.251*	0.230*	0.258*	0.219*	0.195*	0.223*	0.246*
R3	0.275*	0.221*	0.260*	0.236*	0.308*	0.179*	0.243*	0.250*
R4	0.375*	0.276*	0.258*	0.313*	0.318*	0.261*	0.306*	0.305*
R5	0.009	0.051	0.141*	0.048	0.091*	0.105*	0.139*	0.096*
R6	0.087*	0.097*	0.110*	0.091*	0.085*	0.102*	0.093*	0.120*

Note: R1-R6 corresponds to the six recommendations released by the world health organization. They are as follows. **R1**: Regularly wash my hands with soap for at least 20 seconds, **R2**: Cover my nose and mouth when coughing or sneezing, **R3**: Keep a distance of at least 1 meter from other people, **R4**: Avoid shaking hands, hugging or kissing when greeting others, **R5**: Use alcohol-based hand rub’ and **R6**: Avoid touching my nose, eyes and mouth. Spearman rank correlation test is used for this analysis.

*, and ** denote significance at 1 and 5 percent levels respectively.

Trust could also be a facilitator for adherence [15]. In our study, we see that distrust was lower among those who adhered (14.5%) compared to those who did not adhere to the WHO recommendations (29.3%). Table 5 presents the correlation between level of trust and level of adherence for each WHO recommended preventive measure. Trust in COVID-19 information from the WHO is positively correlated with adherence. The correlation is stronger for hygiene measures (R1, R2) and avoiding physical contact (R3, R4).

**Table 5:** Correlations between level of trust and level of adherence.

Adherence	Trust							
	Germany	United Kingdom	Denmark	Netherlands	France	Portugal	Italy	Overall
R1	0.132*	0.113*	0.122*	0.110*	0.154*	0.041	0.140*	0.116*
R2	0.140*	0.101*	0.133*	0.076**	0.144*	0.075**	0.143*	0.123*
R3	0.126*	0.167*	0.093*	0.110*	0.126*	0.073**	0.080**	0.110*
R4	0.130*	0.198*	0.107*	0.161*	0.111*	0.099**	0.102*	0.128*
R5	0.028	0.058	0.124*	0.000	0.119*	0.046	0.130*	0.081*
R6	0.050	0.049	0.040	-0.001	0.122*	0.056	0.102*	0.061*

Note: R1-R6 corresponds to the six recommendations released by the world health organization. They are as follows. **R1**: Regularly wash my hands with soap for at least 20 seconds, **R2**: Cover my nose and mouth when coughing or sneezing, **R3**: Keep a distance of at least 1 meter from other people, **R4**: Avoid shaking hands, hugging or kissing when greeting others, **R5**: Use alcohol-based hand rub’ and **R6**: Avoid touching my nose, eyes and mouth. Spearman rank correlation test is used for this analysis.

* and ** denote significance at 1 and 5 percent levels respectively.

To further examine if familiarity drives adherence independent of trust, we run ordered logistic regression models of the following econometric specification.

$$Y_{i}=\alpha_{0}+F_{i}\alpha_{1}+T_{i}\alpha_{2}+\varepsilon_{i}$$

where *Y_i_* denotes adherence to each preventive measure, *F_i_* denotes overall familiarity, *T_i_* denotes trust in WHO information on COVID-19 and *ε_i_* corresponds to the random error term. Robust standard errors were used in all regressions.

**Fig 3:**
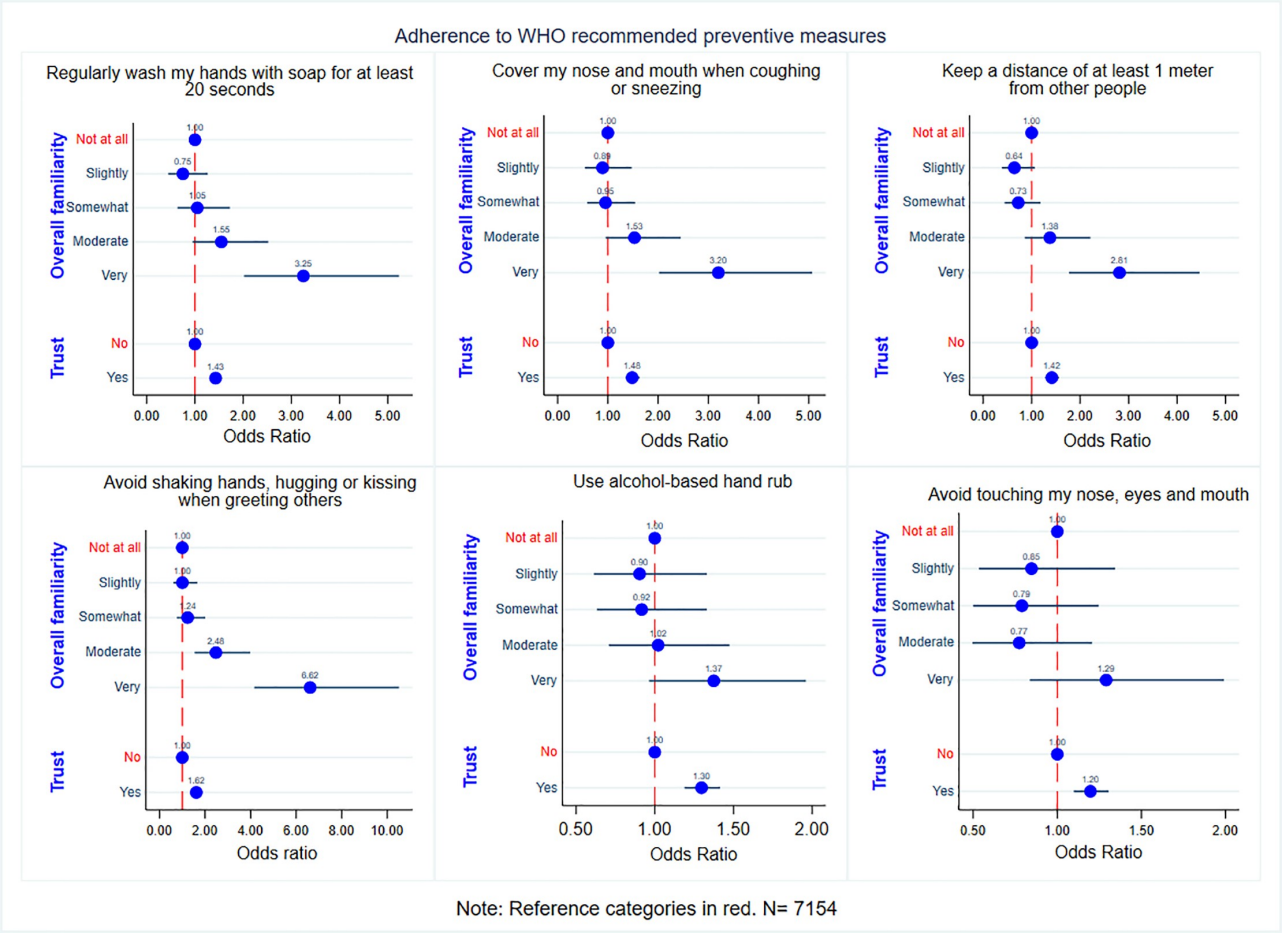
Odds ratio for adherence to WHO recommended preventive measures by overall familiarity and trust in information from the WHO.

Fig 3 presents a graphical description of the Odds Ratio (OR) and 95% Confidence Interval (CI) for the relationship between overall familiarity and adherence while simultaneously controlling for trust in information from the WHO. Our findings strengthen the evidence from simple correlations. Controlling for trust, being very familiar compared to not at all familiar increases the likelihood of adherence with statistical significance retained only for hygiene measures (p<0.001 for R1 and R2) and avoiding physical contact (p<0.001 for R3 and R4). Trust in information from the WHO is also positively related to adherence (p<0.001 for R1-R6).

As a robustness check, we also recode our familiarity variable to a binary measure (familiar or not). Respondents choosing any response other than “very familiar” are considered to be not familiar with WHO recommendations or otherwise. We repeat our analysis with this categorization to ensure enough observations in each group to carry out a robust analysis. Controlling for trust, being familiar compared to not familiar increases the likelihood of adherence for all preventive measures (p <0.001 for R1-R6). Trust in information from the WHO is also positively related to adherence (p<0.001 for R1-R6). Results are robust when controlling for respondent’s perception of others adherence of WHO recommendations, which has been shown to have a sizeable effect on one’s own adherence [16]. The relationship also holds for all countries separately (results are available from the authors on request). The results indicate that both overall familiarity and trust is positively related to adherence independent of one another. We also notice that the effect size for familiarity is larger than that for trust (almost 2 times larger for R1-R4), highlighting a potential larger influence of the familiarity variable in driving adherence.

Overall, at first sight this would mean familiarity implies adherence and trust is a catalyst for this relationship. However, the relationship between familiarity, adherence and trust is not so direct. Factors such as the severity of the COVID-19 crisis and other perceived worries could be influencing each of these factors independently and together. For instance, respondents from Italy and Portugal reported the highest levels of familiarity and adherence, but at the same time showed diverging profiles on COVID-19 attributed death counts (Table 1) and trust in WHO information during the period of our study. In Italy, adherence to physical distancing recommendations is as high as in Portugal, although Italy reports lower trust compared to Portugal. That this high adherence in Italy is narrowed to only physical distancing measures could be attributed to the necessity of adherence given the severity of the pandemic (Table 1). However, Portugal still tops adherence in all measures (including hygiene), which could be facilitated by the high levels of trust in information. This is suggestive of the ability of the WHO to act without any coercion when there are high levels of trust, especially when adherence corresponds to recommendations that are difficult to enforce socially or legally such as hand hygiene.

## Discussion

The ongoing threat to global health from COVID-19 poses critical challenges to governments, medical communities, health organizations, businesses and the public in responding to the evolving pandemic. With an abundance of misinformation on the disease, governments and health organizations need to be meticulous in disseminating up to date and evidence-based information to the public. The guidelines and recommended preventive behaviors as put forward by WHO and other national level public health agencies is of immense importance given the increasing prevalence of cases and emergence of new variants. The WHO mainly recommends hygiene and physical contact precautions to the public given that coronavirus is mainly transmitted through droplets and aerosols. As the COVID-19 vaccination process has just begun in many countries and we still have a long way to go before achieving herd immunity on a global scale, the importance of non-pharmaceutical interventions such as social distancing, use of protective equipment such as face masks and other hygiene behaviors in containing the coronavirus needs to be highlighted [17]. Given that the pandemic is still ongoing, we used data from a pan European survey collected in April 2020 to evaluate the efficiency and effectiveness of the risk communication strategies put in place by the WHO so far during the pandemic. Following are some insights and attention points on risk communication as learned from our findings.

First, our survey results suggest that overall familiarity and adherence with the recommendations is quite high in most countries in Europe. This indicates both the effectiveness of the WHO risk communication strategy and the interest among the public to seek and follow better practices. Countries reporting high levels of familiarity (Portugal and Italy) were also the top adherers. Similarly, countries reporting lower levels of familiarity (UK, Netherlands and Germany) performed worse on adherence. Although there could be other factors influencing this relationship, our results suggest that increasing familiarity with preventive measures could lead to higher levels of adherence among the public, and hence is an effective way to help contain and mitigate the spread of infectious diseases.

Second, we observe considerable heterogeneity in adherence to the different recommendations. Overall, people complied better with avoiding physical contact, but less with hand hygiene and avoiding touching eyes, nose or mouth. Both sets of recommendations involve behavioral modifications with the exception that during the first stage of the pandemic, social distancing was legally and socially enforced, which could be one explanation for the higher adherence rates. Literature also shows that non-adherence to be high especially when recommendations involve behavioral modifications [18]. Given that countries are moving back and forth between lockdown and exit strategies and the simultaneous warnings from the WHO on further waves of coronavirus transmission (or emergence of new variants of coronavirus) [19], it is crucial that the public keep up with social distancing measures even when not legally enforced.

Although social distancing measures has been mostly recommended given the nature of coronavirus transmission, hand washing is also important given that there could be indirect transmission via infected surfaces [17]. However, hand washing has a lower adherence rate globally given the complex interaction of many behavioral aspects that drives compliance to hand hygiene [20]. Hence, there is an increased need to put higher emphasis on improving adherence to hand hygiene and, most importantly, designing policies to ensure that adherence to social distancing does not fade off without legal enforcement over time.

Third, our analysis suggests evidence for heterogeneities in adherence based on socio-demographic characteristics of the respondents. Particularly, we find older, female, and higher educated respondents to report higher levels of familiarity and adherence. Additionally, we also find those respondents with vulnerable household members to have higher levels of adherence. Therefore, steps should be taken to increase awareness among the groups that are less likely to be familiar with or adhere to the preventive measures, in particular the young, males, less educated and households with non-vulnerable family members, since they also play a role in transmitting the virus.

Older people, who are more vulnerable to COVID-19, report higher levels of familiarity and adherence. Possibly they seek more information, or risk communication has been tailored to them better. But, it is equally important to increase awareness among younger people about the risks of not adhering to recommendations, because even if they themselves are less vulnerable, as potential carriers of the virus they may infect others who are. Similarly, households that do not have a vulnerable family member might be less worried about getting infected and hence show poor adherence. Literature shows higher levels of adherence among women in general, attributing this to several factors including early cognitive maturation, capacity for self-care and the stronger perceived need to comply to social expectations [21]. Higher educated respondents might have higher levels of health literacy, which is required to critically assess the information provided in relation to their behaviors [22]. Thus, we might conclude that risk messages may not fit to all groups alike and, therefore, need to be customized to the specific risks and concerns in that group.

Fourth, trust in information from the WHO could influence adherence to its recommendations. Trust is an overlooked aspect in crisis management [23]. Public health organizations need to be more transparent and receptive in their communication to gain the trust of citizens. Most importantly, if the severity of the pandemic in a country is high, this could imply that trust levels are already low, and people are more worried. Hence strategies to improve both adherence and trust should take into consideration the severity of the pandemic in the country and the level of worries among the population. Finally, low trust in authoritative bodies could also be associated with low interpersonal trust (perception of the adherence of others to WHO recommendations) in the society as a whole [24], resulting in covid induced worries, social fear or acts of self-interest such as panic buying and stock piling, which makes crisis management more difficult. Having a perception that others in the community do not adhere to the WHO recommendations could also reduce one’s own level of adherence [25]. Thus, during these hard times, risk communication should not miss out on messages that could improve the public’s trust in their community members and organizations that provide credible information.

Our findings are based on data collected during the first wave of the pandemic. This was the initial and escalating phase of COVID-19, characterized by panic and worries attributed to limited knowledge of COVID-19. Lessons from the first wave of COVID-19 may help all stake holders to be better prepared for further waves of the pandemic, especially with regards to risk communication which is a key element of outbreak control strategy. Identification of subgroup populations that are less likely to trust, to be familiar or adhere signals the need to tailor risk communication strategies that is different from what was implemented in the first wave. However, measures that were effective in the first wave may not necessarily prove to be always successful in future. Pandemic fatigue, misalignment of individual and collective benefits and/or shifts in government response may bring about changes to public adherence over time. Our results from the initial wave of COVID-19 also provides the ground for building a longitudinal dataset to understand changes in public adherence over time and a robust evidence for factors driving these changes, which will in turn lead to “precision policy planning and evaluation” [26].

Finally, we address some limitations of our study. First, the online nature of our survey may generate a sampling bias in our results. Particularly, all respondents may not have an equal chance of being invited to participate in the survey depending on their level of online engagement. Access to internet may be further correlated with age and socio-economic status. To reduce this bias, Dynata employs a multi-source panel recruitment model which uses a wide range of sources to reach respondents, thereby ensuring a diverse population to participate in the survey. For example, Dynata collaborates with loyalty programs which allow to survey people who would normally never participate in online research. They then bring all these sources together on one integrated platform, thereby ensuring total control over the exact blend composition of the respondents. Also, studies show internet based questionnaires to be a “suitable alternative” to more traditional survey methods [27, 28]. Second, if the WHO risk communication activities are primarily web-based, this might bias our results as the offline population may not have access to this information. However, the WHO has been active in implementing strategies to ensure that the message reaches everyone. For instance, the WHO has collaborated with the International Telecommunication Union (ITU) to send texts to public on preventive measures against COVID-19 [29]. Moreover, the WHO has also set in place the Risk Communication and Community Engagement (RCCE) system to provide guidance for countries in implementing effective strategies during COVID-19 such as public communication, community engagement and capacity building, all of which may further help to engage with masses that are otherwise difficult to reach [1]. Third, we assume that the participant’s response to the familiarity question reflects the same level of familiarity for each WHO recommendation (given that we show the poster before asking the question). Nevertheless, it is possible that this may not be the case and respondent’s answers may be biased in a way that a higher level of familiarity with one (or more) recommendation may dominate low levels of familiarity with other recommendation or vice-versa. Finally, we emphasize that the level of trust in information from the WHO corresponds to COVID-19 information in general and trust in WHO recommended preventive measures may constitute only a part of this information. Therefore, we assume that the respondents have the same level of trust in all of the COVID-19 related information from the WHO and also the same level of trust in each of the WHO recommended preventive measure.

## Conclusion

Overall, we find that information from WHO in the context of COVID-19 is well trusted and acted upon by the public. However, our results suggest the need to strengthen efforts to reach the less vulnerable parts of the population in information campaigns, and to take the worries of the public into account in the design and dissemination of risk communication strategies. Furthermore, our findings call for efforts to get regular feedback from the public on their familiarity with the most recent recommendations and their support for policy measures that increase compliance with these recommendations. As both the pandemic and the recommendations evolve, risk communication needs to be tailored to the different groups in society in order to be more effective.

## Supporting information

S1 Table Classification of level of education into low/medium/high categories. (DOCX)

S1 Appendix Survey in all languages. (DOCX)

## List of abbreviation

WHO: World Health Organization
