# Supplementary material for: Risk communication during COVID-19: A descriptive study on familiarity with, adherence to and trust in the WHO preventive measures
Source: PLoS One. 2021 Apr 29;16(4):e0250872. doi: 10.1371/journal.pone.0250872 (PMC8084201; doi:10.1371/journal.pone.0250872)
Supplement: S1 Table — (DOCX) [file pone.0250872.s001.docx]

| **S1 Table: Classification of level of education into low/medium/high categories** | | |
| --- | --- | --- |
| **Country** | **Level of education** | **Category** |
| United Kingdom | Combined Junior and Infant School/ Infant School | Low |
|  | Junior School |  |
|  | Comprehensive School |  |
|  | Comprehensive School (GCSE)/ Secondary Modern (GCSE)/ Grammar School (GSCE)/ City Technology College | Medium |
|  | College and Institution of Higher education | High |
|  | Open College -College of Technology - Institute/ Teacher Training College |  |
|  | University/ Open University |  |
| Portugal | Sem Estudos | Low |
|  | Primário Incompleto |  |
|  | Primário Completo |  |
|  | Nível Médio Incompleto | Medium |
|  | Nível Médio Completo |  |
|  | Superior Incompleto | High |
|  | Superior Completo |  |
| Netherlands | LO (lagere school, LAVO, VGLO) | Low |
|  | LBO (LBO, LTS, ITO, LEAO, Huishoudschool, LLO) |  |
|  | MAO (MAVO, IVO, MULO, ULO, 3jr HBS, 3jr VWO, 3jr VHMO) |  |
|  | MBO (MTS, UTS, MEAO) | Medium |
|  | HAO (HAVO, VWO, Atheneum, Gymnasium, NMS, HBS, Lyceum) |  |
|  | HBO (HTS, HEAO, Wetensch. kand., Univers. onderwijs kand.) | High |
|  | WO (Universitair onderwijs, Doctoraalopleiding, TH) |  |
| Italy | Scuola elementare | Low |
|  | Scuola media inferiore |  |
|  | Istituto professionale | Medium |
|  | Scuola superiore |  |
|  | Università | High |
|  | Master |  |
|  | Dottorato |  |
| Germany | Grundschule | Low |
|  | Hauptschule |  |
|  | Realschule |  |
|  | Gymnasium/ Berufliches Gymnasium/ Fachgymnasium, Gesamtschule | Medium |
|  | Fachoberschule, Fachschule, Berufsschule, Berufsfachschule |  |
|  | Technische Hochschule, Pädagogische Hochschule, Kunsthochschule/ Musikhochschule | High |
|  | Fachhochschule |  |
|  | Universität, Technische Universität |  |
| France | École Primaire | Low |
|  | Collège |  |
|  | Lycée d`Enseignement général et technologique | Medium |
|  | Lycée professionnel |  |
|  | Grande École de Commerce et de Gestion/ scientifique, Établissement d`Enseignement supérieur catholique/ artistique/ agricole | High |
|  | Grand Établissement/ École normale supérieure/ d`Ingénieur/ d`Architecture/ nationale vétérinaire |  |
|  | Université, Institut universitaire de Technologie/ national polytechnique/ d`Études politiques/ universitaire de Formation des Maîtres |  |
| Denmark | Folkeskolen - f.eks. 9. eller 10. klasse | Low |
|  | Gymnasial uddannelse - f.eks. Almen Gymnasium, HHX, HTX osv. | Medium |
|  | En videregående erhvervsuddannelse - f.eks. landbrugs-, social- og sundheds uddannelser, produktionsskole | High |
|  | En mellemlang videregående uddannelse |  |
|  | Universitets uddannelse |  |
