# Supplementary material for: Risk communication during COVID-19: A descriptive study on familiarity with, adherence to and trust in the WHO preventive measures
Source: PLoS One. 2021 Apr 29;16(4):e0250872. doi: 10.1371/journal.pone.0250872 (PMC8084201; doi:10.1371/journal.pone.0250872)
Supplement: S1 Appendix — (DOCX) [file pone.0250872.s002.docx]

S1 Appendix: Survey in all languages

Project: Countering COVID-19: A European Survey on the acceptability of and commitment to preventive measures.

Country: United Kingdom

Consent form

Dear Participant, 
Thank you for participating in this survey on the novel coronavirus (COVID-19). The goal of this study is to understand people's attitudes towards the disease and the associated risks. Please answer the survey to the best of your knowledge and abilities.

 Before you start, please:
 

• Make sure you have about 20 minutes of uninterrupted time;
• Maximize your browser window;
• Switch your phone to a silent mode;
• Switch off your e-mail, phone notifications and anything else that may distract you.
 
Please, do not use external sources of information like the Internet to search for information. Many people may not know the answers to some questions, but please answer every question according to your belief if you are not sure what the right answer is.


Your data will be treated in accordance with the provisions of the European Data Protection Regulation (GDPR EU).


 
 **CONSENT FORM**
 I consent to participate in this survey. I understand that all data will be kept confidential by the researcher. My personal information will not be stored with the data. I am free to withdraw at any time, without giving a reason.

- I voluntarily consent to participate in this study.
- I do not wish to participate.

**Block: Demographics**

**Q1** How old are you currently?

**Q2** What is your gender?

- Male
- Female

**Q3** Does your household include any of the following members (other than you)?

- Very young children and babies
- Children
- Disabled person(s)
- Someone with diagnosed chronic medical conditions (such as heart or lung conditions or diabetes)
- Elderly person(s)
- ⊗None of the ones mentioned above

**Block: Trust**

**Q4**  On the scale below, please indicate to which extent you trust the information from the following sources in the context of COVID-19 situation.

|  | Not at all | Very much |
| --- | --- | --- |

|  | 1 | 2 | 3 | 4 | 5 |
| --- | --- | --- | --- | --- | --- |

| Your national government | 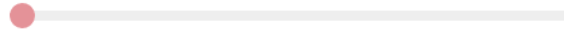 |
| --- | --- |
| The European Union | 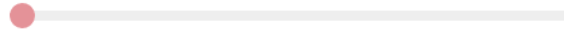 |
| Main national news channels / newspapers | 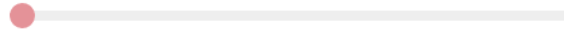 |
| Social media (Facebook, Twitter, Instagram) | 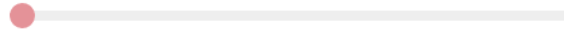 |
| Hospitals | 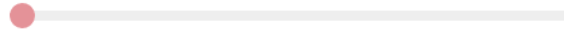 |
| General practitioner/ Family doctor | 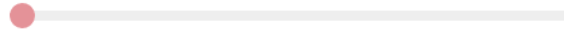 |
| World Health Organization (WHO) | 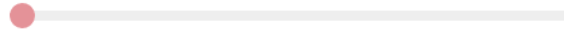 |
| Your relatives and friends | 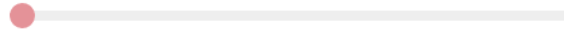 |

**Block: Familiarity and compliance with WHO recommendations**

Now we would like you to carefully read the 5 basic protective measures against the novel coronavirus recommended by the World Health Organization (WHO).


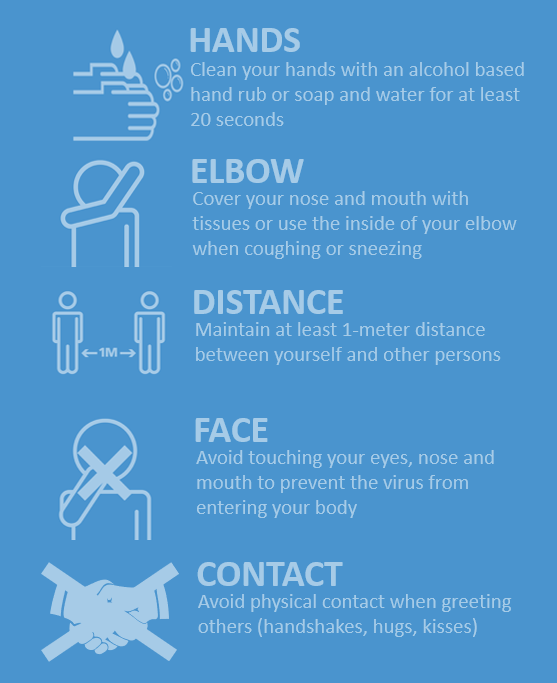


**Q5** How familiar were you with these basic protective measures against the novel coronavirus before you saw the poster?

- Not at all familiar
- Slightly familiar
- Somewhat familiar
- Moderately familiar
- Very familiar

**Q6** Next, we would like to know about your own practices related to the novel coronavirus.
 Thinking about the last four weeks, did you adhere to the following activities due to concerns about the novel coronavirus?

|  | No | Yes, a bit | Yes, quite strongly | Yes, fully |
| --- | --- | --- | --- | --- |
| Regularly wash my hands with soap for at least 20 seconds |  |  |  |  |
| Cover my nose and mouth when coughing or sneezing |  |  |  |  |
| Keep distance of at least 1 meter from other people |  |  |  |  |
| Avoid shaking hands, hugging or kissing when greeting others |  |  |  |  |
| Use alcohol-based hand rub |  |  |  |  |
| Avoid touching my nose, eyes and mouth |  |  |  |  |

**Q7** In your opinion, do others in your community adhere to the WHO basic protective measures against the novel coronavirus these days?

|  | No | Yes, a bit | Yes, quite strongly | Yes, fully |
| --- | --- | --- | --- | --- |
| Regularly wash their hands with soap for at least 20 seconds |  |  |  |  |
| Cover their nose and mouth when coughing or sneezing |  |  |  |  |
| Keep distance of at least 1 meter from other people |  |  |  |  |
| Avoid handshakes, kisses and hugs when greeting others |  |  |  |  |
| Use alcohol-based hand rub |  |  |  |  |
| Avoid touching their eyes, nose and mouth |  |  |  |  |

*************************************************************************************

#

Country: Germany

**Consent**
Liebe Teilnehmerin, lieber Teilnehmer,
Vielen Dank für Ihre Teilnahme an dieser Umfrage zum neuartigen Coronavirus (COVID-19). Ziel dieser Studie ist es, die Einstellung der Menschen zu dieser Erkrankung und den damit verbundenen Risiken zu verstehen. Bitte beantworten Sie die Umfrage nach bestem Wissen und Gewissen.

 Bevor Sie beginnen, bitte:
  
 • Stellen Sie sicher, dass Sie ungefähr 20 Minuten ununterbrochene Zeit haben.
 • Maximieren Sie Ihr Browserfenster.
 • Schalten Sie Ihr Telefon in einen lautlosen Modus.
 • Schalten Sie Ihre E-Mails, Telefonbenachrichtigungen und alles andere aus, was Sie ablenken könnte.
  
 Bitte verwenden Sie keine externen Informationsquellen wie das Internet, um nach Informationen zu suchen. Viele Menschen kennen die Antworten auf einige Fragen möglicherweise nicht, bitte beantworten Sie jede Frage einfach nach bestem Wissen und Gewissen, wenn Sie nicht sicher sind, welche Antwort die richtige ist.
  
Ihre Daten werden gemäß den Bestimmungen der Europäischen Datenschutzverordnung Grundverordnung (DSGVO EU) behandelt.
 
**Einverständniserklärung**

 Ich bin damit einverstanden, an dieser Umfrage teilzunehmen. Ich verstehe, dass alle Daten vom Forscher vertraulich behandelt werden. Meine persönlichen Informationen werden nicht mit den Daten gespeichert. Ich kann die Teilnahme ohne Angabe von Gründen zurückziehen.

- Ich stimme freiwillig zu an dieser Umfrage teilzunehmen
- Ich möchte nicht teilnehmen

**Block: Demographics**

**Q1** Wie alt sind Sie heute?

**Q2** Bitte nennen Sie ihr Geschlecht

- Männlich
- Weiblich

**Q3** Umfasst Ihr Haushalt eines der folgenden Mitglieder (Sie nicht mitgezählt)?

- Kleinkinder und Neugeborene
- Kinder
- Körperlich oder geistig behinderte Menschen
- Jemand mit diagnostizierten chronischen Erkrankungen (wie z.B. Herz- oder Lungenerkrankungen oder Diabetes)
- Ältere Person (en)
- ⊗Keine der oben genannten

**Block: Trust**

**Q4** Geben Sie auf der folgenden Skala bitte an, inwieweit Sie Informationen aus den folgenden Quellen im Zusammenhang mit der COVID-19-Situation vertrauen. Informationen von

|  | Überhaupt nicht | Sehr |
| --- | --- | --- |

|  | 1 | 2 | 3 | 4 | 5 |
| --- | --- | --- | --- | --- | --- |

| der deutschen Bundesregierung | 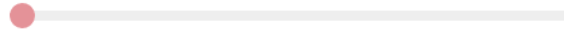 |
| --- | --- |
| der europäischen Union | 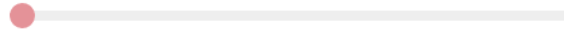 |
| den großen Nachrichtensendern und Zeitungen | 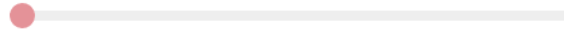 |
| sozialen Medien (Facebook, Twitter, Instagram) | 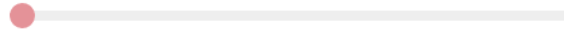 |
| Krankenhäusern | 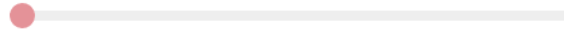 |
| Ihrem/r Allgemeinmediziner(in)/ Hausarzt/ Hausärztin | 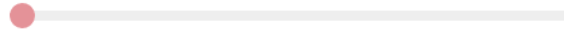 |
| der Weltgesundheitsorganisation (WHO) | 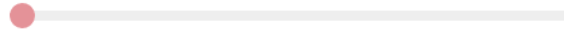 |
| Ihrer Familie / Ihren Freunden | 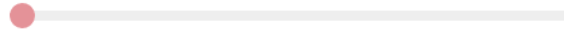 |

**Block: Familiarity and compliance with WHO recommendations**

Nun möchten wir Sie bitten, die 5 grundlegenden von der Weltgesundheitsorganisation (WHO) empfohlenen Schutzmaßnahmen gegen das neuartige Coronavirus sorgfältig zu lesen.

**
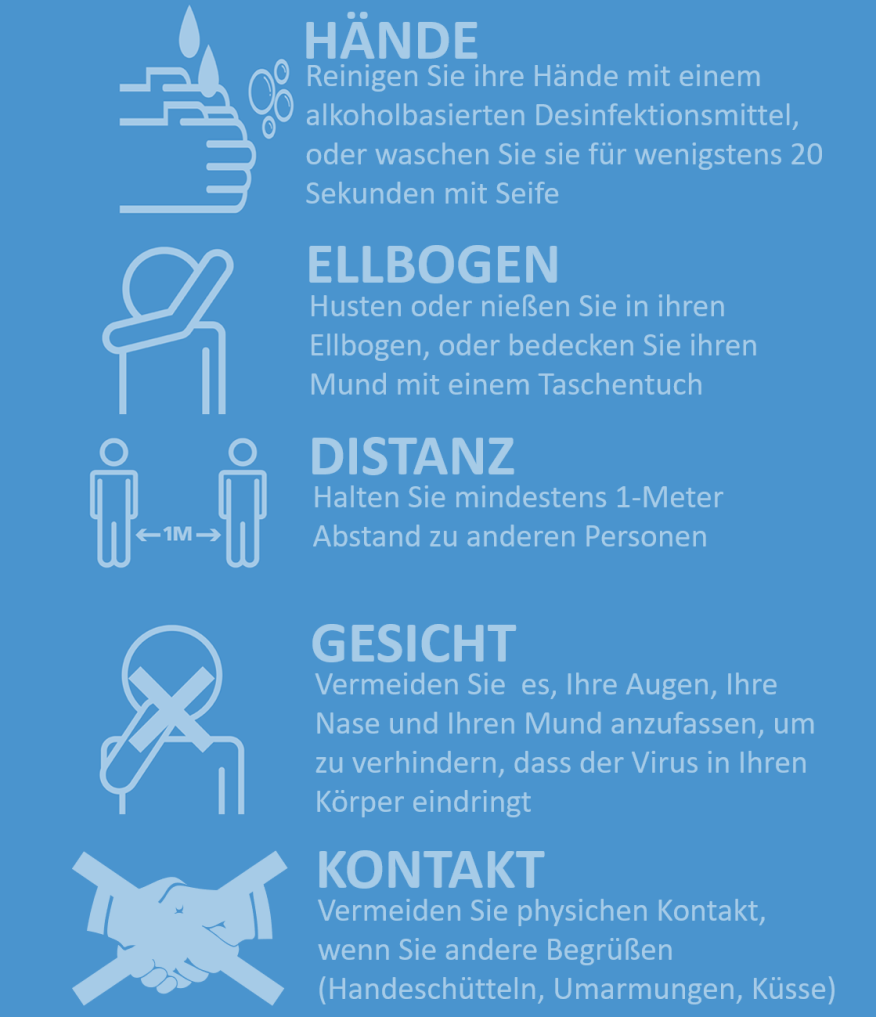
**

**Q5** Wie vertraut waren Sie mit diesen grundlegenden Schutzmaßnahmen gegen das neuartige Coronavirus, bevor Sie das Poster sahen?

- Überhaupt nicht vertraut
- Ein wenig vertraut
- Etwas vertraut
- Mäßig vertraut
- Sehr vertraut

**Q6** Als nächstes möchten wir Sie über Ihre eigenen Verhaltensweisen im Zusammenhang mit dem neuartigen Coronavirus befragen.
 Wenn sie an die letzten vier Wochen denken, haben Sie aufgrund von Bedenken hinsichtlich des neuartigen Coronavirus folgende Verhaltensweisen an den Tag gelegt?

|  | Nein | ja, manchmal | Ja, häufig | Ja, immer |
| --- | --- | --- | --- | --- |
| Sich regelmäßig die Hände mindestens 20 Sekunden mit Seife waschen |  |  |  |  |
| Die Nase und Mund beim Husten oder Niesen bedecken |  |  |  |  |
| Einen Abstand von mindestens 1- Meter zu anderen Personen einzuhalten |  |  |  |  |
| Es vermeiden Hände zu schütteln, zu umarmen oder zu küssen, wenn man andere begrüßt |  |  |  |  |
| Die Verwendung von alkoholbasiertem Handdesinfektionsmittel |  |  |  |  |
| Es vermeiden die eigene Nase, die Augen und den Mund zu berühren |  |  |  |  |

**Q7** Halten sich Ihrer Meinung nach andere in Ihrer Gemeinde im Moment an die grundlegenden Schutzmaßnahmen der WHO gegen das neuartige Coronavirus?

|  | Nein | ja, manchmal | Ja, häufig | Ja, immer |
| --- | --- | --- | --- | --- |
| Sich regelmäßig die Hände mindestens 20 Sekunden mit Seife waschen |  |  |  |  |
| Die Nase und Mund beim Husten oder Niesen bedecken |  |  |  |  |
| Einen Abstand von mindestens 1- Meter zu anderen Personen einzuhalten |  |  |  |  |
| Es vermeiden Hände zuschütteln, zu umarmen oder zu Küssen, wenn man andere begrüßt |  |  |  |  |
| Die Verwendung von alkoholbasiertem Handdesinfektionsmittel |  |  |  |  |
| Es vermeiden die eigene Nase, die Augen und den Mund zu berühren |  |  |  |  |

*************************************************************************************

#

**Country: Denmark**

**Consent**
Kære deltager, 
Tak fordi du vil deltage i denne undersøgelse om den nye coronavirus (COVID-19). Formålet med undersøgelsen er at forstå folks holdning til sygdommen og de tilhørende risici. Besvar venligst spørgsmålene efter din bedste evne.

Før du begynder, vil vi bede dig om at:
 

• Sørge for at du forbliver uforstyrret de næste 20 minutter;
• Maksimere dit browservindue;
• Sætte din telefon på lydløs;
• Slukke for din e-mail, telefonbeskeder og alt andet, der kan distrahere dig.
 
Benyt venligst ikke eksterne informationskilder som internettet til at søge efter oplysninger. Mange mennesker kender måske ikke svarene på alle spørgsmål, men besvar venligst alle spørgsmål i henhold til hvad du tror, selvom du ikke er sikker på, hvad det rigtige svar er.


Din data vil blive behandlet i henhold til den europæiske databeskyttelsesforordning (GDPR).


 
 **SAMTYKKE**
Jeg giver hermed samtykke til at deltage i undersøgelsen. Jeg er indforstået med, at data vil blive behandlet og opbevaret fortroligt af forskerne. Mine personoplysninger gemmes ikke sammen med de svar jeg afgiver. Mit samtykke kan til enhver tid trækkes tilbage uden at give en grund for dette.

- Jeg giver hermed samtykke til, at deltage i denne undersøgelse
- Jeg ønsker ikke at deltage

**Block: Demographics**

**Q1** Hvor gammel er du?

**Q2** Hvad er dit køn?

- Mand
- Kvinde

**Q3** Indeholder din husstand nogle af følgende (udover dig):

- Meget unge børn eller
- Børn
- Handicappede
- Personer diagnosticeret med kroniske lidelser (såsom hjerte- eller lungesygdomme eller diabetes)
- Ældre personer
- ⊗Ingen af ovennævnte

**Block: Trust**

**Q4** På skalaen nedenfor skal du angive, i hvilket omfang du har tillid til oplysningerne fra følgende kilder i forbindelse med COVID-19-situationen.

|  | Slet ikke | I høj grad |
| --- | --- | --- |

|  | 1 | 2 | 3 | 4 | 5 |
| --- | --- | --- | --- | --- | --- |

| Regeringen | 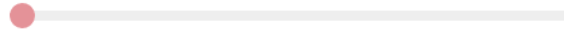 |
| --- | --- |
| Den Europæiske Union | 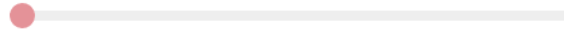 |
| Nationale nyhedskanaler og aviser | 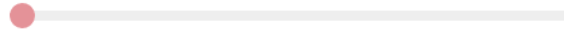 |
| Sociale medier (Facebook, Twitter, Instagram) | 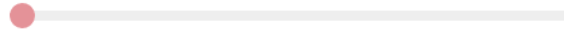 |
| Hospitaler | 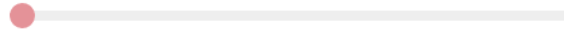 |
| Praktiserende læger | 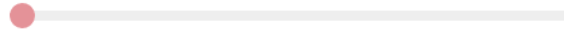 |
| Verdenssundhedsorganisationen (WHO) | 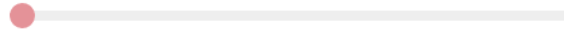 |
| Din familie og venner | 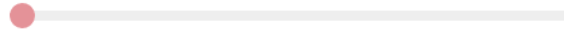 |

**Block: Familiarity and compliance with WHO recommendations**

Vi vil nu bede dig om grundigt at læse de 5 grundlæggende beskyttelsesforanstaltninger mod den nye coronavirus, som er anbefalet af Verdenssundhedsorganisationen (WHO).

**
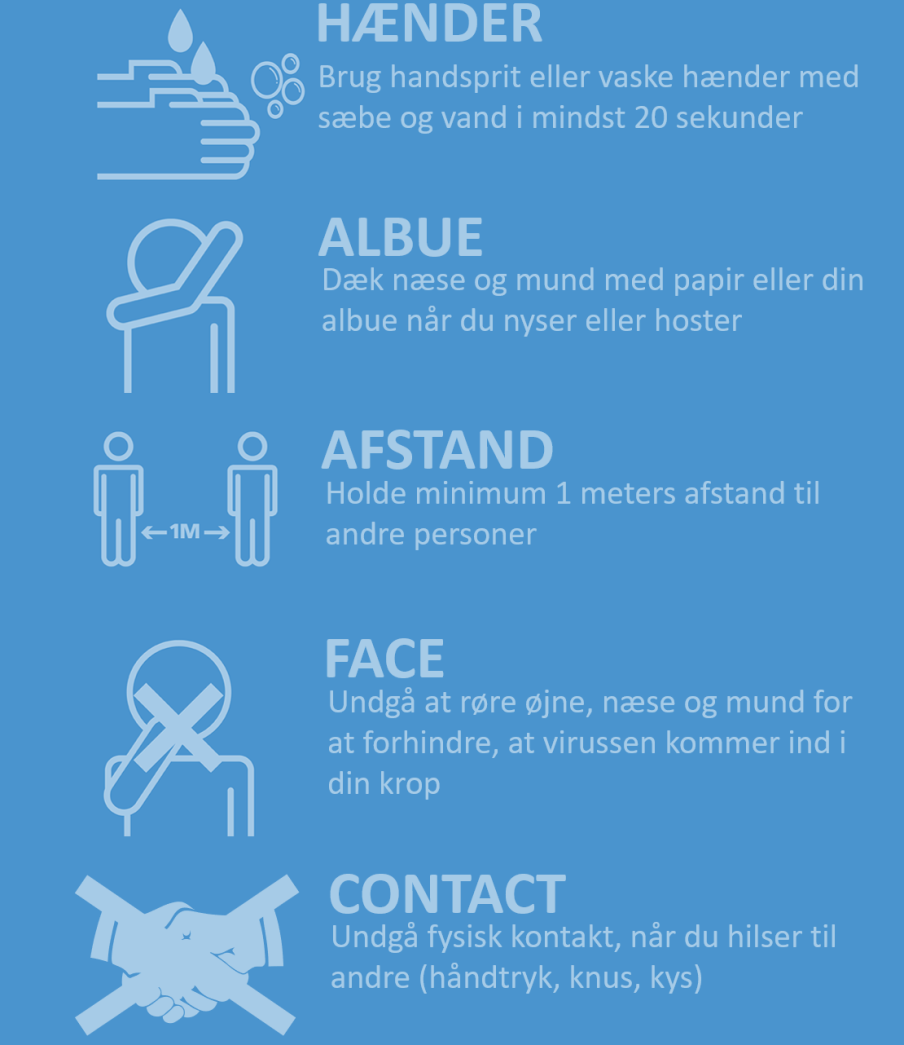
**

**Q5** Hvor bekendt var du i forvejen med disse grundlæggende beskyttelsesforanstaltninger mod den nye coronavirus, før du så plakaten?

- Ingen kendskab
- Lidt kendskab
- Kendskab til en hvis grad
- Moderat kendskab
- Stort kendskab

**Q6** Vi vil nu gerne vide lidt om din egen forholdsregler i forbindelse med den nye coronavirus.
 Tænk tilbage på de sidste 4 uger, overholdt du følgende aktiviteter grundet bekymring over den nye coronavirus?

|  | Nej | Ja, en smule | Ja, i høj grad | Ja, til fulde |
| --- | --- | --- | --- | --- |
| Vasker mine hænder regelmæssigt med sæbe i mindst 20 sekunder |  |  |  |  |
| Dækker min mund og næse når jeg hoster eller nyser |  |  |  |  |
| Holde minimum 1 meters afstand til andre mennesker |  |  |  |  |
| Undgår at give hænder, kramme eller kysse når jeg hilser på andre |  |  |  |  |
| Bruger handsprit |  |  |  |  |
| Undgår at røre min næse, øjne og mund |  |  |  |  |

**Q7** Efter din mening, overholder andre i dit lokalsamfund WHO's grundlæggende beskyttelsesforanstaltninger mod den nye coronavirus i disse dage?

|  | Nej | | Ja, en smule | Ja, i høj grad | Ja, til fulde |
| --- | --- | --- | --- | --- | --- |
| Vasker deres hænder regelmæssigt med sæbe i mindst 20 sekunder | |  |  |  |  |
| Dækker deres mund og næse når de hoster eller nyser | |  |  |  |  |
| Holde minimum 1 meters afstand til andre mennesker | |  |  |  |  |
| Undgår at give hænder, kramme eller kysse når de hilser på andre | |  |  |  |  |
| Bruger håndsprit | |  |  |  |  |
| Undgår at røre deres øjne, næse og mund | |  |  |  |  |

**Country: France**

**Consent**

Cher\Chère participant(e),

Merci pour votre participation à cette enquête sur le nouveau coronavirus (COVID-19). Le but de cette étude est de comprendre les attitudes de la population envers le virus et les risques associés. Veuillez répondre au sondage du mieux que vous le pouvez sur la base de vos connaissances actuelles.

 Avant de commencer, nous vous prions de :


 • Vous assurer que vous disposez d'environ 20 minutes continues devant vous ;
 • Maximiser la fenêtre de votre navigateur ;
 • Mettre votre téléphone en mode silencieux ;
 • Désactiver vos e-mails, notifications téléphoniques et autres sources de distraction.


Veuillez ne pas utiliser de sources d'informations externes comme internet pour rechercher des informations. Beaucoup de personnes ne connaîtront peut-être pas les réponses à certaines questions, mais nous vous demandons de répondre à chaque question dans l'état de vos connaissances, ou de vos croyances si vous n'êtes pas sûr(e) de la bonne réponse.

Vos données seront traitées conformément aux dispositions du règlement européen sur la protection des données (RGPD UE).


 **FORMULAIRE DE CONSENTEMENT**

J'accepte de participer à cette enquête. Je comprends que toutes les données seront gardées de manière confidentielle par le chercheur. Mes informations personnelles ne seront pas conservées avec les données. Je suis libre de me retirer de l'enquête à tout moment, sans donner de raisons.

- Je consens volontairement à participer à cette étude.
- Je ne souhaite pas participer.

**Block: Demographics**

**Q1** Quel âge avez-vous ?

**Q2** Êtes-vous :

- Un homme
- Une femme

**Q3** Est-ce que le ménage dans lequel vous vivez comprend l'un des membres suivants (autre que vous-même) ?

- Enfants en bas âge et nourrissons
- Enfants
- Personne(s) handicapée(s)
- Personne(s) souffrant d'une condition médicale chronique diagnostiquée (condition cardiaque, des poumons ou diabète)
- Personne(s) âgée(s)
- ⊗Aucune des catégories mentionnées ci-dessus

**Block: Trust**

**Q4** Sur l'échelle ci-dessous, veuillez indiquer le degré de confiance que vous avez dans les sources d'information suivantes dans le contexte de l'épidémie de COVID-19.

|  | Pas du tout | Beaucoup |
| --- | --- | --- |

|  | 1 | 2 | 3 | 4 | 5 |
| --- | --- | --- | --- | --- | --- |

| Votre gouvernement | 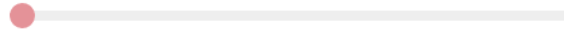 |
| --- | --- |
| L'Union Européenne | 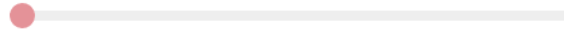 |
| Les chaines d'information et journaux nationaux principaux | 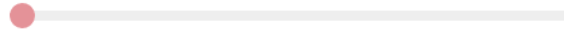 |
| Les réseaux sociaux (Facebook, Twitter, Instagram) | 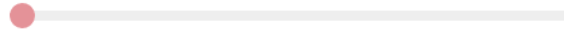 |
| Les hôpitaux | 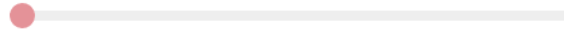 |
| Les médecins généralistes | 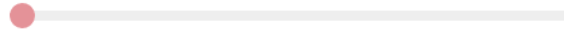 |
| L'Organisation Mondiale de la Santé (OMS) | 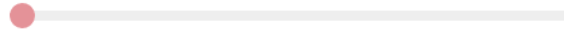 |
| Vos proches et amis | 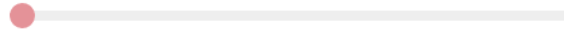 |

**Block: Familiarity and compliance with WHO recommendations**

A présent, nous souhaiterions que vous lisiez attentivement les 5 mesures préventives de base contre le nouveau coronavirus recommandées par l'Organisation Mondiale de la Santé (OMS).


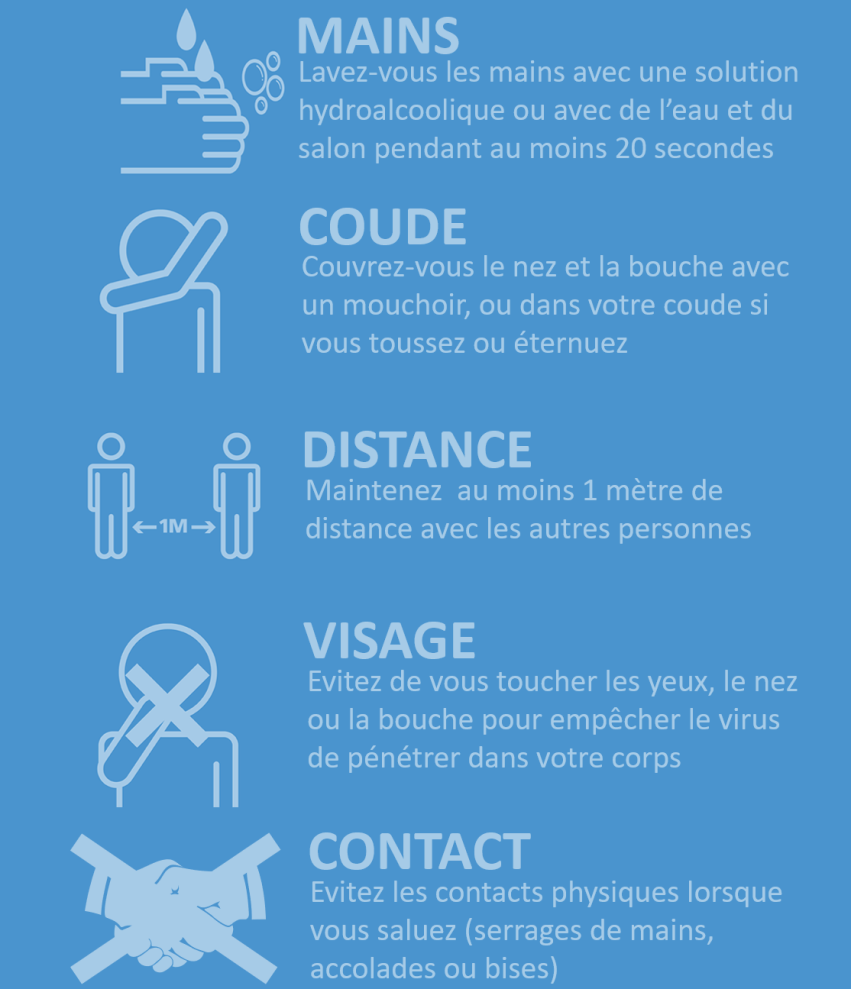


**Q5** Connaissiez-vous bien les mesures préventives de base contre le nouveau coronavirus, avant de voir le poster ?

- Pas du tout
- Un peu
- Relativement
- Modérément
- Très bien

**Q6** Ensuite, nous aimerions connaitre vos propres habitudes en lien avec le nouveau coronavirus.

Au cours des quatre dernières semaines, avez-vous mis en place les gestes suivants en raison des inquiétudes liées au nouveau coronavirus ?

|  | Non | Oui, un peu | Oui, plutôt fortement | Oui, complètement |
| --- | --- | --- | --- | --- |
| Se laver les mains régulièrement avec du savon pendant 20 secondes |  |  |  |  |
| Se couvrir le nez et la bouche quand je tousse ou j'éternue |  |  |  |  |
| Garder une distance d'au moins 1 mètre avec les autres personnes |  |  |  |  |
| Éviter de se serrer la main, de donner des accolades ou de faire la bise pour se saluer |  |  |  |  |
| Utiliser une solution hydroalcoolique pour les mains |  |  |  |  |
| Éviter de se toucher le nez, les yeux et la bouche |  |  |  |  |

**Q7** Selon vous, les personnes de votre entourage respectent-elles les mesures préventives de base contre le nouveau coronavirus en ce moment ?

|  | Non | | Oui, un peu | Oui, plutôt fortement | Oui, complètement |
| --- | --- | --- | --- | --- | --- |
| Se laver régulièrement les mains avec du savon pendant au moins 20 secondes | |  |  |  |  |
| Se couvrir le nez et la bouche losqu'on tousse ou éternue | |  |  |  |  |
| Garder une distance d'au moins 1 mètre avec les personnes présentant des symptômes semblables à la grippe | |  |  |  |  |
| Éviter de se serrer la main, de donner des accolades et de faire la bise pour se saluer | |  |  |  |  |
| Utiliser une solution hydroalcoolique pour les mains | |  |  |  |  |
| Éviter de se toucher les yeux, le nez et la bouche | |  |  |  |  |

**Country: Netherlands**

**Consent**
Beste deelnemer,
Bedankt voor uw deelname aan deze enquête over het nieuwe coronavirus (COVID-19). Het doel van deze studie is om de houding van mensen ten opzichte van de ziekte en de bijbehorende risico's te begrijpen. Beantwoord de enquête naar uw beste weten en kunnen.
 
Voordat u begint:
 
• Zorg ervoor dat u ononderbroken ongeveer 20 minuten de tijd heeft;
• Maximaliseer uw browservenster;
• Zet uw telefoon in een stille modus;
• Schakel uw e-mail, telefoonmeldingen en al het andere dat u zou kunnen afleiden uit.
 
Gebruik alstublieft geen externe informatiebronnen, zoals internet, om naar informatie te zoeken. Het kan zijn dat u het antwoord van een vraag niet weet, maar probeer de vraag te beantwoorden met wat u denkt dat het juiste antwoord is, ook al weet u dat niet zeker.
Uw gegevens worden behandeld in overeenstemming met de bepalingen van de algemene verordening gegevensbescherming (GDPR EU).
 
 
**TOESTEMMINGSFORMULIER**
 
Ik geef toestemming om deel te nemen aan deze enquête. Ik begrijp dat alle gegevens door de onderzoeker vertrouwelijk worden behandeld. Mijn persoonlijke gegevens worden niet opgeslagen. Ik kan me op elk moment terugtrekken zonder opgaaf van redenen.

- Ik neem vrijwillig deel aan deze studie.
- Ik neem niet deel aan deze studie.

**Block: Demographics**

**Q1** Hoe oud bent u?

**Q2** Wat is uw geslacht?

- Man
- Vrouw

**Q3** Bestaat uw huishouden uit een of meerdere personen uit de volgende categorieën (los van uzelf)?

- Zeer jonge kinderen en baby's
- Kinderen
- Gehandicapte / beperkte personen
- Iemand met gediagnosticeerde chronische medische aandoeningen (zoals hart- of longaandoeningen of diabetes)
- Oudere personen
- ⊗Geen van bovenstaande

**Block: Trust**

**Q4** Geef per item hieronder aan of u de bron vertrouwt of niet, wat betreft COVID-19.

|  | Helemaal niet | Heel erg |
| --- | --- | --- |

|  | 1 | 2 | 3 | 4 | 5 |
| --- | --- | --- | --- | --- | --- |

| Rijksoverheid (RIVM) | 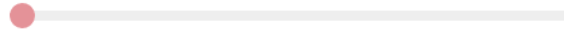 |
| --- | --- |
| Europese Unie | 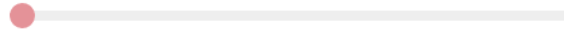 |
| Belangrijkste nieuwszenders / kranten | 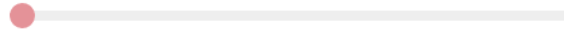 |
| Sociale media (Facebook, Twitter, Instagram) | 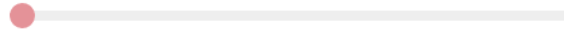 |
| Ziekenhuizen | 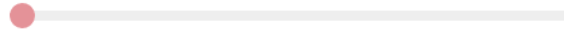 |
| Huisarts | 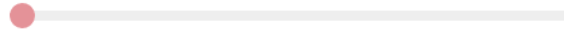 |
| Wereldgezondheidsorganisatie (World Health Organization) | 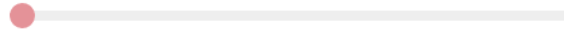 |
| Vrienden en familie | 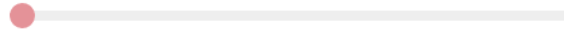 |

**Block: Familiarity and compliance with WHO recommendations**

We zouden graag willen dat u nu de 5 maatregelen tegen het coronavirus aandachtig doorneemt, aangeraden door de Wereldgezondheidsorganisatie (WHO).


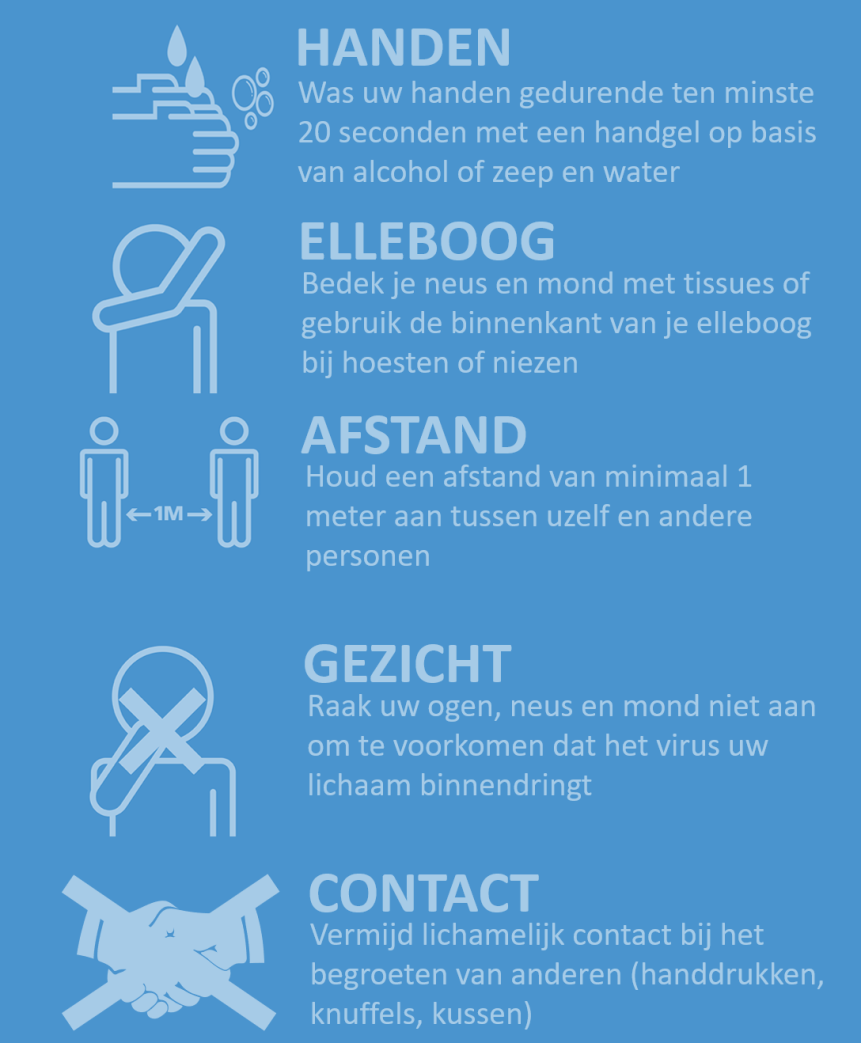


**Q5** In hoeverre was u bekend met de maatregelen tegen het coronavirus voordat u de poster zag?

- Helemaal niet bekend
- Een beetje bekend
- Enigszins bekend
- Redelijk bekend
- Zeer bekend

**Q6** We willen nu graag wat meer weten over uzelf met betrekking tot het coronavirus. Gebaseerd op de afgelopen vier weken, hield u zich aan de volgende maatregelen vanwege de zorgen over het coronavirus?

|  | Nee | | Ja, een beetj | Ja, voornamelijk | Ja, volledig |
| --- | --- | --- | --- | --- | --- |
| Regelmatig mijn handen wassen met zeep gedurende minstens 20 seconden | |  |  |  |  |
| Mijn neus en mond bedekken bij hoesten of niezen | |  |  |  |  |
| Houd tenminste 1 meter afstand van andere mensen | |  |  |  |  |
| Het vermijden van handen schudden, knuffelen of zoenen bij het begroeten van anderen | |  |  |  |  |
| Het gebruik van desinfecterende handgel | |  |  |  |  |
| Het vermijden van mijn neus, mond en ogen aanraken | |  |  |  |  |

**Q7** Houden anderen in uw omgeving zich aan de voorzorgsmaatregelen tegen het coronavirus, denkt u?

|  | Nee | Ja, een beetje | Ja, voornamelijk | Ja, volledig |
| --- | --- | --- | --- | --- |
| Regelmatig hun handen wassen met zeep voor ten minste 20 seconden |  |  |  |  |
| Het bedekken van de neus en mond bij hoesten en/of niezen |  |  |  |  |
| Houd tenminste 1 meter afstand van andere mensen |  |  |  |  |
| Het vermijden van handen schudden, zoenen en knuffelen bij het begroeten van anderen |  |  |  |  |
| Het gebruik van desinfecterende handgel |  |  |  |  |
| Het vermijden van hun ogen, neus en mond aanraken |  |  |  |  |

*************************************************************************************

**Country: Italy**

**Consent**

Gentile Partecipante,

Grazie per aver partecipato a questo sondaggio sul nuovo coronavirus (COVID-19). L'obiettivo di questo studio è comprendere le opinioni delle persone nei confronti della malattia e dei rischi ad essa associati. Ti preghiamo di rispondere al sondaggio al meglio delle tue conoscenze e abilità.
Prima di iniziare, ti preghiamo di:
 

• Assicurarti di avere circa 20 minuti di tempo ininterrotto;
• Massimizzare la finestra del browser;
• Mettere il telefono in modalità silenziosa;
• Disattivare l'e-mail, le notifiche telefoniche e qualsiasi altra cosa che possa distrarti.
 

 Ti preghiamo di non usare fonti esterne di informazioni come Internet per cercare informazioni. Molte persone potrebbero non saper rispondere ad alcune domande, ma ti invitiamo a rispondere a tutte le domande in base alla tue convinzioni se non sei sicuro di quale sia la risposta giusta.
  
 I tuoi dati saranno trattati in conformità alle disposizioni del regolamento europeo sulla protezione dei dati *(European Data Protection Regulation, GDPR EU).*
 
 
 **MODULO DI CONSENSO INFORMATO**

Acconsento a partecipare a questo sondaggio. Comprendo che tutti i dati saranno mantenuti riservati dal ricercatore. Le mie informazioni personali non verranno archiviate insieme ai dati. Sono libero di ritirarmi in qualsiasi momento, senza fornire alcuna motivazione.  Acconsento volontariamente a partecipare a questo sondaggio. Non desidero partecipare.

- Acconsento volontariamente a partecipare a questo sondaggio
- Non desidero partecipare.

**Block: Demographics**

**Q1** Quanti anni hai?

**Q2** Sesso

- Maschio
- Femmina

**Q3** La composizione della tua famiglia include uno dei seguenti soggetti (diversi da te)?

- Bambini molto piccoli e/o neonati
- Bambini
- Persona(e) disabile(i)
- Qualcuno con patologie croniche diagnosticate (come patologie cardiache o polmonari, o diabete)
- Persona(e) anziana(e)
- ⊗Nessuno di quelli sopra menzionati

**Block: Trust**

**Q4** Nella scala sottostante, ti chiediamo di indicare fino a che punto ti fidi delle informazioni dalle seguenti fonti nel contesto della situazione COVID-19.

|  | Per niente | Molto |
| --- | --- | --- |

|  | 1 | 2 | 3 | 4 | 5 |
| --- | --- | --- | --- | --- | --- |

| Il tuo governo nazionale | 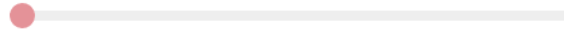 |
| --- | --- |
| L'Unione Europea | 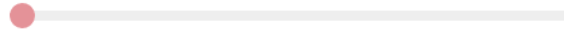 |
| I principali canali di notizie / giornali nazionali | 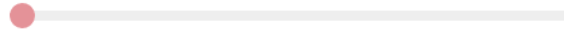 |
| Social media (Facebook, Twitter, Instagram) | 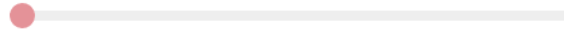 |
| Ospedali | 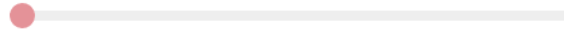 |
| Medico Generale/Medico di famiglia | 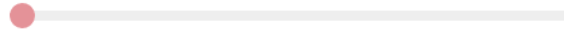 |
| Organizzazione mondiale della sanità (OMS) | 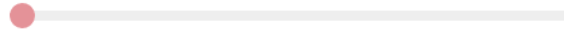 |
| I tuoi parenti e amici | 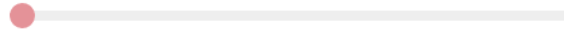 |

**Block: Familiarity and compliance with WHO recommendations**

Ora ti preghiamo di leggere attentamente le 5 misure di protezione di base contro il nuovo coronavirus raccomandate dall'Organizzazione Mondiale della Sanità (OMS).


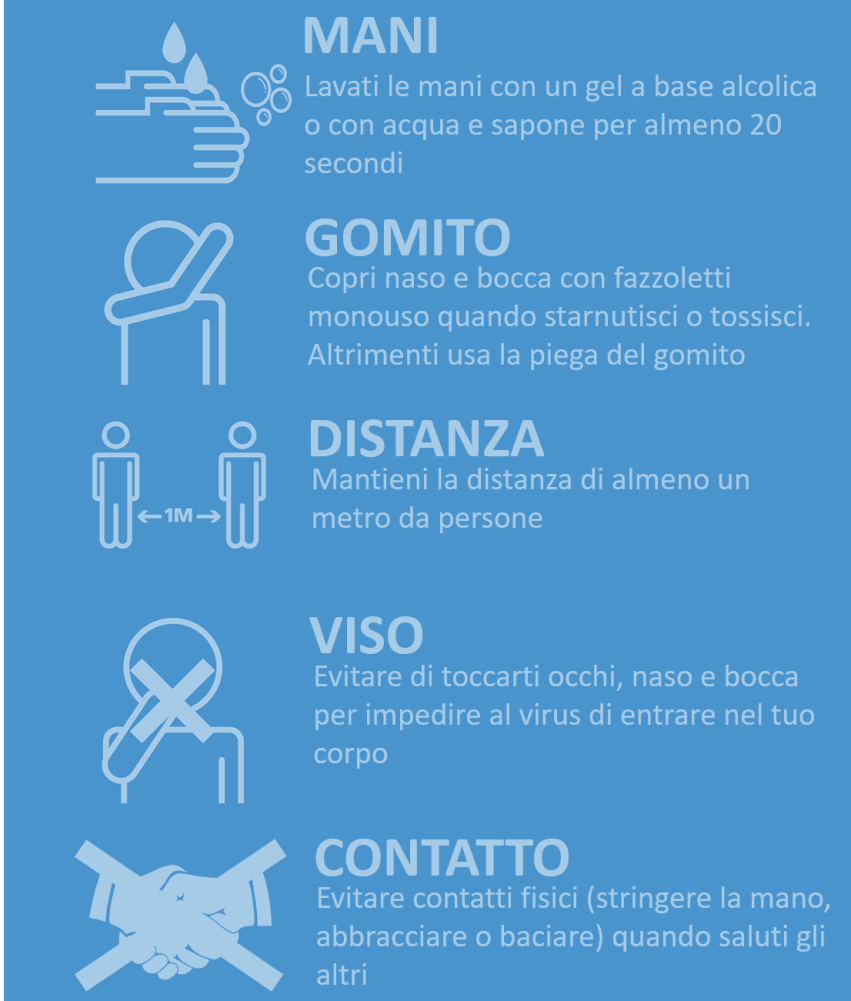


**Q5** Quanto eri a conoscenza di queste misure di protezione di base contro il nuovo coronavirus prima di vedere il poster?

- Per niente
- Leggermente
- Abbastanza
- Moderatamente
- Molto

**Q6** Successivamente, vorremmo conoscere le tue abitutine relative al nuovo coronavirus.

Pensando alle ultime quattro settimane, hai aderito alle seguenti attività a causa delle preoccupazioni per il nuovo coronavirus?

|  | No | Sì, un po' | Sì, abbastanza fortemente | Sì, pienamente |
| --- | --- | --- | --- | --- |
| Lavare regolarmente le mani con sapone per almeno 20 secondi |  |  |  |  |
| Coprirti il ​​naso e la bocca quando tossisci o starnutisci |  |  |  |  |
| Mantenere una distanza di almeno 1 metro dalle altre persone |  |  |  |  |
| Evitare di stringere la mano, abbracciare o baciare quando saluti gli altri |  |  |  |  |
| Usare il gel igienizzante a base alcolica |  |  |  |  |
| Evitare di toccarti naso, occhi e bocca |  |  |  |  |

**Q7** Secondo te, altri nella tua comunità aderiscono alle misure di protezione di base dell'OMS contro il nuovo coronavirus in questi giorni?

|  | No | Sì, un po' | Sì, abbastanza fortemente | Sì, completamente |
| --- | --- | --- | --- | --- |
| Lavare regolarmente le mani con sapone per almeno 20 secondi |  |  |  |  |
| Coprirsi il ​​naso e la bocca quando tossiscono o starnutiscono |  |  |  |  |
| Mantenere una distanza di almeno 1 metro dale altre persone |  |  |  |  |
| Evitare strette di mano, baci e abbracci quando salutano gli altri |  |  |  |  |
| Usare il gel igienizzante a base alcolica |  |  |  |  |
| Evitare di toccarsi gli occhi, il naso e la bocca |  |  |  |  |

*************************************************************************************

**Country: Portugal**

**Consent**

Caro Participante,

Obrigado por participar neste inquérito sobre o novo coronavírus (COVID-19). O objetivo deste estudo é entender as atitudes das pessoas em relação à doença e os riscos associados. Por favor, responda ao questionário da melhor forma possível com os seus conhecimentos.

 Antes de começar, por favor:
    
 • Verifique se tem cerca de 20 minutos de tempo ininterrupto;
 • Maximize a janela do seu navegador;
 • Coloque o seu telefone no modo silencioso;
 • Desative o seu e-mail, notificações por telefone e qualquer outra coisa que possa distraí-lo.
    
Por favor, não use fontes externas de informação como a Internet para procurar informações. Muitas pessoas podem não saber as respostas a algumas perguntas, mas responda a todas as perguntas de acordo com a sua opinião, mesmo se não tiver certeza de qual é a resposta certa.

Os seus dados serão tratados de acordo com as disposições do Regulamento Europeu de Proteção de Dados (GDPR EU).
 **FORMULÁRIO DE CONSENTIMENTO**

Autorizo ​​participar neste inquérito. Entendo que todos os dados serão mantidos em sigilo pelo investigador. As minhas informações pessoais não serão armazenadas com os dados. Sou livre de terminar a qualquer momento, sem dar uma razão.

- Voluntariamente consinto em participar neste estudo.
- Não desejo participar.

**Block: Demographics**

**Q1** Quantos anos tem atualmente?

**Q2** Qual é o seu género?

- Masculino
- Feminino

**Q3** A sua família inclui algum dos seguintes membros (além de si)?

- Crianças e bebés muito jovens
- Crianças
- Pessoas com deficiência
- Alguém com condições médicas crónicas diagnosticadas (como problemas cardíacos ou pulmonares ou diabetes)
- Idoso (s)
- ⊗Nenhuma das mencionadas acima

**Block: Trust**

**Q4** Na escala abaixo, indique até que ponto confia nas informações das seguintes fontes no contexto da situação da COVID-19.

|  | Nada | Muito |
| --- | --- | --- |

|  | 1 | 2 | 3 | 4 | 5 |
| --- | --- | --- | --- | --- | --- |

| Governo nacional | 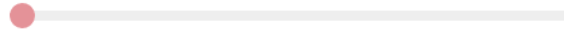 |
| --- | --- |
| União Europeia | 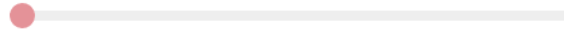 |
| Principais canais noticiosos / jornais nacionais | 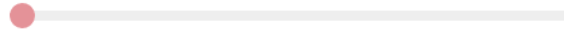 |
| Redes sociais (Facebook, Twitter, Instagram) | 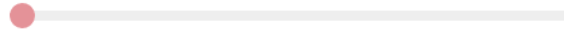 |
| Hospitais | 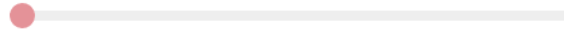 |
| Clínico geral / Médico de família | 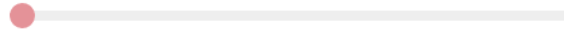 |
| Organização Mundial da Saúde (OMS) | 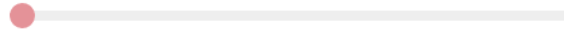 |
| Seus parentes e amigos | 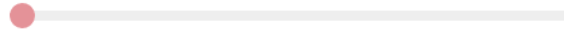 |

**Block: Familiarity and compliance with WHO recommendations**

Agora gostaríamos que lesse atentamente as 5 medidas básicas de proteção contra o novo coronavírus recomendadas pela Organização Mundial da Saúde (OMS).


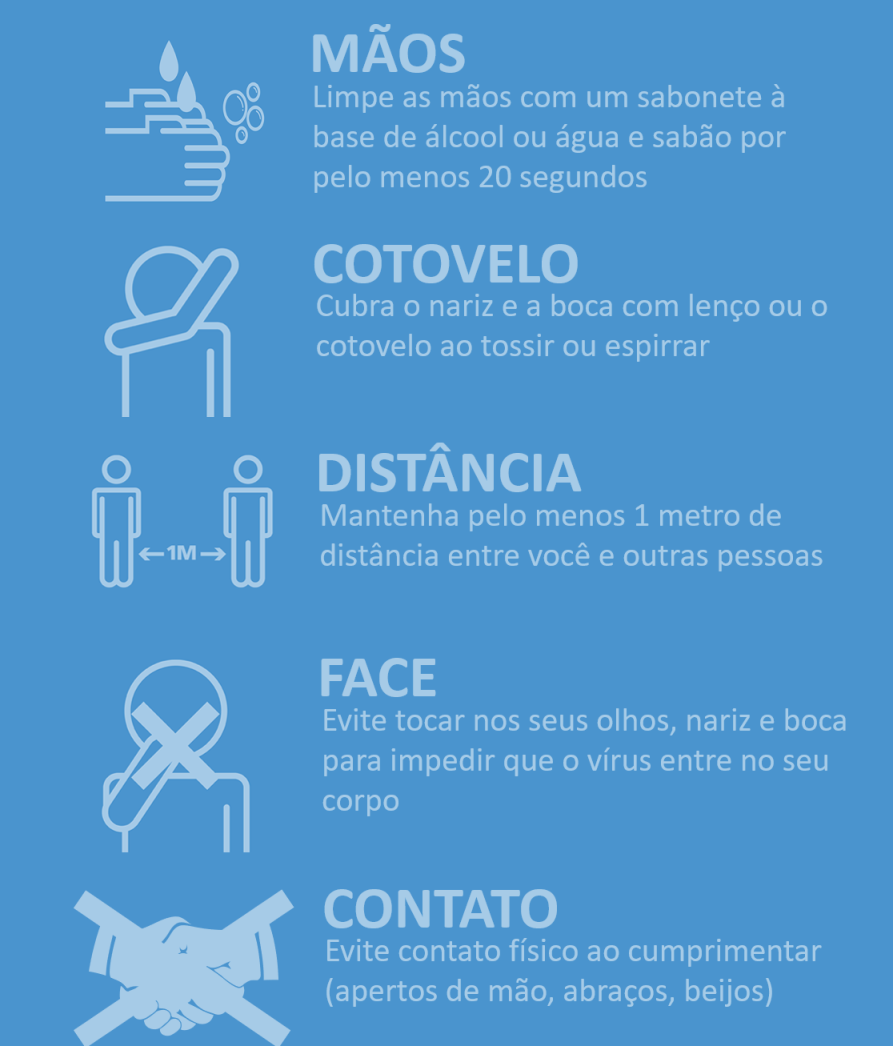


**Q5** Estava familiarizado com essas medidas básicas de proteção contra o novo coronavírus antes de ver o póster?

- Não familiar
- Pouco familiar
- Um pouco familiar
- Moderadamente familiar
- Muito familiar

**Q6** Em seguida, gostaríamos de saber sobre suas próprias práticas relacionadas com o novo coronavírus.
Pensando nas últimas quatro semanas, aderiu às seguintes atividades devido a preocupações com o novo coronavírus?

|  | Não | Sim, um pouco | Sim, bastante | Sim, totalmente |
| --- | --- | --- | --- | --- |
| Lavar as mãos regularmente com sabão por pelo menos 20 segundos |  |  |  |  |
| Cobrir o nariz e a boca quando tossir ou espirrar |  |  |  |  |
| Manter a distância de pelo menos 1 metro de outras pessoas |  |  |  |  |
| Evitar apertar as mãos, abraçar ou beijar ao cumprimentar os outros |  |  |  |  |
| Usar detergente para as mãos à base de álcool |  |  |  |  |
| Evitar tocar meu nariz, olhos e boca |  |  |  |  |

**Q7** Na sua opinião, outras pessoas da sua comunidade aderem às medidas básicas de proteção da Organização Mundial da Saúde (OMS) contra o novo coronavírus atualmente?

|  | Não | Sim, um pouco | Sim, bastante | Sim, totalmente |
| --- | --- | --- | --- | --- |
| Lavar regularmente as mãos com sabão por pelo menos 20 segundos |  |  |  |  |
| Cobrir o nariz e a boca ao tossir ou espirrar |  |  |  |  |
| Manter distância de pelo menos 1 metro de outras pessoas |  |  |  |  |
| Evitar apertos de mão, beijos e abraços ao cumprimentar os outros |  |  |  |  |
| Usar detergentes para as mãos à base de álcool |  |  |  |  |
| Evitar tocar nos olhos, nariz e boca |  |  |  |  |

*************************************************************************************
